# How value is enacted by public actors within urban development funding and the implications for levelling up in England

**DOI:** 10.1177/26349825231155880

**Published:** 2023-03-10

**Authors:** Andrew Barnfield

**Affiliations:** University of Bristol, UK

**Keywords:** Value, urban development, performativity, diagram, space

## Abstract

The aim of the article is to understand how public actors perform value and how this effects urban development. This article answers this by examining how value is enacted in urban development funding in England. Urban development funding from central to local government is a key source of financing for regeneration and infrastructure projects. Attending to how value is enacted reveals who shapes the underpinning rationale, which in turn influences how value is represented in decision-making and urban development projects. Building on the recent work in geography that questions the concept of value and drawing on performativity and diagrammatic thinking, this article argues that HM Treasury is central to how value is enacted and that the prevailing conception of value in urban development prioritises short-term, financial outcomes despite an awareness of the need to be sensitive to temporal and spatial concerns. The findings proceed from 24 in-depth interviews with senior civil servants, politicians, local government representatives and policy experts that took place during the summer of 2021. The article concludes by considering the implications for enacting value under the UK government’s Levelling up agenda.

## Introduction: Enacting value

The aim of this article is to understand how public actors in England enact value and how this effects urban development. Central government funding for urban development in England is an important resource for local and combined authorities^1^ (Roberts, 2020). The current funding streams available in England include the Towns Fund, a £3.6 billion fund for the regeneration of high streets and the Levelling up fund with £4.8 billion available for infrastructure projects (HM Government, 2022). The urban development projects that have received levelling up money in 2021 include £19.5m for the regeneration of Rotherham Town Centre (Rotherham Council, 2021), £12.8m for sustainable transport improvements through Gloucester city centre (Gloucestershire County Council, 2021) and £15.5m for the renovation of the Moseley Road Baths and Balsall Heath library in Birmingham (Birmingham City Council, 2021).

Urban scholars have examined the financialisation of urban development and real estate practices by studying the interactions between investor, developer, and planners to draw attention to how investors’ idea of value is employed via regulatory and fiscal reforms, strengthening their role as drivers of spatial development (Aalbers, 2019; Weber, 2002, 2010). Within geography, and the social sciences more broadly, there has been a growing concern with the concept of value. This has been led by a disquiet with market value and its absorption of ecological or communal value (Andueza, 2021; Kay and Kenney-Lazar, 2017; Mell et al., 2016; Laruffa, 2021; Pérez and Herrera, 2020). The Covid-19 pandemic instigated a consideration of what kinds of cities, spaces and practices will be or need to be valued in the future (Cresswell, 2021; McFarlane, 2021).

The recent work from geography has, to date, paid limited attention to the enactment or performativity of value. Studies of performing value in real estate (Crosby and Henneberry, 2016; Robin, 2018; Weber, 2016) have drawn on notions of performativity from science and technology studies through the work of Michel Callon (2010) to think through how the networks of expert knowledge, technical devices, and regulations are implicated in the shaping of urban space and decision-making. Performativity is a useful concept because it enables the examination of how value is generative of spaces, places and practices (Weber, 2021). Beneficial too is the work of Nathalie Heinich (2017), who has drawn on French pragmatic sociology to provide the groundwork to focus on the study of the processes of value. Heinich (2020) argues for a consideration of how value is developed and comprehended by public actors through the contexts, subjects and objects. Heinich describes this as the ‘three moments’ of value.

In this article, I add to these approaches by drawing from an understanding of performativity from non-representational geographies (Simpson, 2020; Thrift, 2008). I do so for three reasons. First, non-representational accounts of performativity do more than increase the array of processes and practices to which to attend. It animates efforts to utilise more of the performative character of the process of knowledge production (McCormack, 2009). This entails thinking of techniques of knowledge production (e.g. guidance documents, appraisal rules, and evaluation criteria) as performative agents and not as devices of representational capture that suspend the ‘enacting’. Second, relatedly, a diagram becomes a device that brings forth a reality that is yet to come, a new type of reality, and offers a new point of view for government documents and frameworks (Deleuze, 2014 [1986]). Third, non-representational accounts of performativity offer a dynamic, enlivened conception of space. This is space not as a static container within which things happen, rather space as an ongoing contingent and contextual process, emergent through a range of practices and processes (McCormack, 2014).

This article adds to the literature from geography, urban development and performativity by examining how value is enacted by public actors in urban development funding in England. This is important because how value is (re)produced by actors and institutions shapes everyday geographies and spaces of urban development. The article is based on 24 in-depth interviews with public actors (civil servants, local government officials and policy experts) in England who are involved with the administering, assessing or allocating of urban development funding. The key finding from this article is that HM Treasury (HMT) has an influential position in how value is understood and enacted, and that the prevailing conception of value in urban development prioritises short-term, financial outcomes despite an awareness of the need to be sensitive to temporal and spatial concerns.

This article is set out as follows: the next section explores literature from geographical interactions with value, how the study of value in urban development has highlighted the roles/rules for certain parts of the industry, and the non-representational qualities of diagrams for thinking about frameworks and guidelines. The following section introduces the case study and how urban development funding works in England. Then, I explore how value is enacted via HMT,^2^ UK government documents, and interviews with public actors highlighting four key aspects: (1) HMT is an influential force for how value is enacted; (2) change will be a slow, incremental process; (3) there is a recognised need to promote more than financial benefits; and (4) public actors are aware that to enact value differently, changes to structures of decision-making need to be brought forth. The article concludes by considering the implications for enacting value under the UK government’s Levelling up^3^ agenda.

### Value as a fulcrum of associations and practices: The spatiality of value, performing value in real estate, and non-representational diagrams

The study of value from a geography and urban development perspective stresses how value shapes the world through spatial practices, the drawing together of objects, tools, ideas and people, and foregrounds the background processes that create urban space. Recent work from urban development and real estate has emphasised how studying value from a performativity perspective enables research that unpacks value as a fulcrum of associations that underpins the built environment.

#### Spatialising value

Value is a concept that geographers have returned, to think through its implication in the forging of a manifold of associations and animating a variety of relations (Pérez and Herrera, 2020). Complication arises between various terms that are employed: value, values and valuation. This stems, in part, from different valuation regimes (the rules and modes of comparison) operating and even competing ‘rather than the concept of value itself’ (Bigger and Robertson, 2017: 69). By value, this article is interested in value as a concept that underpins decisions and procedures in urban development funding. It is a concept of value that emerges from the actions, contexts and objects of public actors and institutions (Heinich, 2020). These are not public values which are the beliefs, norms and principles that should arguably guide public administration and policymaking (MacLean and Titah, 2022).

Contemporary work in geography has taken a critical perspective to value to think through the potentially transformative societal and ecological impacts that could emerge (Kay and Kenney-Lazar, 2017; Kenney-Lazar and Kay 2017a, 2017b; Robertson and Wainwright, 2013; Mell et al., 2016; Moore, 2017). Such work on value sees it as a political-economic concept, supported by Marxian-inspired understandings of its sources, production and social distribution. This corresponds to geographers who have written about how capitalism subsumes other values to reproduce itself (e.g. Collard and Dempsey, 2020; Smith, 2010). This line of thinking suggests that the extraction of value (capital accumulation) is arguably the leading mode of sense-making within contemporary society (Castree, 2009; Harvey, 2006). It structures many everyday spaces and practices from the technologies and techniques of international shipping (Levinson, 2016) to regimes and spaces of care (Gordon et al., 2021) to practices of brand (di)association for commodity goods (Ibert et al., 2019). In terms of urban development, cities have embraced financial speculation and extraction via regulatory reforms and private sector–led partnerships. Such real estate markets seek the continual creation of novel forms of value despite concerns over devaluation (Knuth et al., 2019; Merrifield, 2014).

Value, and how it is conceptualised, is an important concept for thinking about how to bring about new worlds and new ways of living (as well as thinking about the spaces and practices that are resistant to processes of change, exploitation, or commodification (Pérez and Herrera, 2020)). The covid-19 pandemic has given rise to reflections on how positive impacts can affect new spatial arrangements, living practices or movements in time and space. For example, Cresswell (2021) proposes the concept of value to explore how different types of mobilities will be valued, encompassing different speeds and styles of travel, arguing that the pandemic has the potential to hold open a series of opportunities to act collectively in the future. Re-valuing quotidian mobility practices such as the commute could lead to new ways of thinking through travel to work and relations with co-workers (Plyushteva, 2019). The same hope for change in value is expressed by McFarlane (2021) who argues for a new politics of value that is premised on a revaluing of density. This would entail a multi-scalar and multi-sectoral challenge for city governance. It is reliant on a change in registers towards different bodies, infrastructure policies and alliances between different citizens, organisations and elected assemblies.

Value can be a concept that is about affirming practices and spaces, one that aims to promote a certain ethos of living that prioritises the ethical and the communitarian. However, Manning (2020) cautions that what tends to get valued at present are things that have certain resonances with structures of affective power and established standards of value. The body of recent geographical engagement has sought to identify the different associations and practices that are involved in the maintenance of any value regime. It emphasises how value is not stable and objective, and is instead emergent from a field of practices, relationships and knowledges.

To understand the role value plays within urban development, what is required is a focus on how value is developed and comprehended by public actors through the three moments of value (contexts, subjects, and objects) (Heinich, 2020). Heinich’s (2020) work explores ‘a concept of value as a combination of mental frames, objective affordances and social institutions’ (p. 77). This entails examining the interplay of the three moments to see how ‘value is produced through the whole set of operations by which a quality is assigned to an object’ (Heinich, 2020: 87). This involves looking at how value is motivated by the affordances that an object offers to valuation, by the collective representation undertaken by actors, and by the possibilities offered by the contexts in which these representations are activated. This article considers Heinrich’s approach of the three moments to understand how value is enacted in a way that influences what is funded by the government and the types of projects pursued by local authorities in England.

#### Enacting value in urban development

The great recession of 2007–2009, led to a critical reappraisal of the implications and implementations of finance across many aspects of daily life (Tooze, 2018). The effects on urban development, the ownership of urban space, and financial strategies for value extraction from urban space have been a particular critical focus (Aalbers, 2019; Guironnet and Halbert, 2014; Halbert and Attuyer, 2016; Peck and Whiteside, 2016; Weber, 2010). This body of work has drawn attention to how the liberalisation of legal and institutional frameworks has increased the participation of global financial investments into the constructing, maintaining and leveraging of urban environments (Fields, 2018; Gotham, 2009; Weber, 2016).

In addition to the underpinning legal and financial structures, there are numerous actors and fields of expertise involved in urban development. This includes engineers, surveyors and planners who have used different knowledge and expertise to (re)shape urban space at different times and in different places (Gross, 1990; Scott, 2008 [1998]). These are not the only actors who are central to urban development; the important roles of other intermediaries such as brokers, consultants and developers have been highlighted for the ways in which they secure financial capital to particular places through the development of urban spaces (Brill, 2022; David and Halbert, 2014; Fernandez et al., 2016; Halbert and Rouanet, 2014; Searle, 2014).

Building on work that draws on performativity, Weber (2016) examines property cycles in Chicago. She argues that cycles are not the product of mis-learned lessons by actors within the sector. Rather, real-estate actors have material incentives to actively enact cycles. A range of actors are embroiled in the performance of cycles, most notably through investment decisions, and this in turn shapes urban space and development. Weber’s work is useful to encourage consideration of how individual actions, institutional processes and calculative devices are interwoven in the performance of property development.

Robin (2018) adopts a performative lens in her work on the re-development of King’s Cross in London to explore how values are enacted by developers. She argues that real estate values are enacted using malleable planning instruments and through the interconnections of public-sector experts and developers, most notably, in the production planning guidelines for the development. Such tools and institutional mechanisms provide the foundation upon which real estate values dictate in decision-making, and allow them to be performed in space, through urban development projects. Robin concludes that the use of performativity enables researchers to unpack how particular economic metaphors and ideas are enacted through rules, tools and sites.

Correspondingly, Brill (2020) investigates the re-development of London’s Silvertown scrutinising planning permission applications to show how frequently hidden corporate strategies shape developers’ role in urban governance. Brill’s work argues that developers can navigate institutional settings and reduce requirements for meaningful public engagement due to sub-centres of control within the private sector rather than any central power. This complicates the picture of power within private-sector development and suggests that a relational approach is the best way to understand how the private sector operates with and against governance structures.

The studying of experts and sector strategies leads to a consideration of how institutional relationships and accepted practices in turn shape cross-sector working and the development of urban space (Robin, 2018). While attention has been paid to how a range of different private-sector actors enact value through professional valuers’ techniques, financial viability appraisals and economic metaphors that shape market practices through representations of value cycles (Christophers, 2014; Crosby and Henneberry, 2016), less attention has been paid to the enacting of value by public actors, particularly those who operate within government funding schemes for urban development. At present, government policy direction is to reduce regional disparities through urban development and infrastructure projects, which means that how value is enacted is crucial.

#### Non-representational diagrams

The importance of relationality, the situated relationships between people, things, concepts, ideas, and places, within non-representational approaches lends itself to research that highlights the way value is enacted (Anderson and Harrison, 2010; Sletto, 2021). It does so because non-representational approaches do not propose knowledge production itself is key to shaping the socio-material world. Rather, they stress the mesh and interaction of tools, technical devices, advisory groups, regulations and laws, governments and organisations contribute to shaping the world in all sorts of ways (Amin and Thrift, 2017). It is a meshwork that is generative of the world rather than merely reflective (Weber, 2021).

The foregrounding of space as an ongoing contingent and contextual process, emergent through a range of practices and processes, is important because it adds to the emphasis that sense-making is dispersed across bodies, spaces and objects (Barnfield, 2018). By looking at the enacting of value by public actors, the intention is to delve into the procedures, practices and rules that underpin urban development and are, in turn, generative of spaces of feeling and experience (Horlings, 2015; Thrift, 2008). While non-representational approaches have employed the concept of performativity through different types of performance practice including dance (Revill, 2004), running (Barnfield, 2018), and parkour (Saville, 2008) to foreground the need to overcome representations as the primary epistemological medium through which knowledge is extracted from the world (McCormack, 2005), it does not propose the eradication of representations. Rather, they are reanimated as active and affective interventions in a world of relations and movements (McCormack, 2009).

One such reanimation is thinking diagrammatically. A Deleuzian (2014 [1986]: 36 and 37)-inspired interpretation of what a diagram can do is influenced by his reading of both Foucault and Spinoza, as well as his work with Felix Guattari. Here a diagram sheds is static, constraining character. It is replaced by a cartography of the immanent and distributive associations of relations of affective force. It is a device for apprehending and instigating action, movement and future realities. The reconceptualization of the potential of a diagram promulgates it as ‘profoundly unstable or fluid, never ceasing to churn up matter and functions in a way likely to create change’ (Deleuze, 2014 [1986]: 35).

Paulo de Assis (2018: 97) explains that the diagram is ‘thus, an abstract machine defined by its informal functions and matter. It does not make a distinction either between content and expression or between discursive and non-discursive formations. In this sense, it makes it possible to conceive the transformations that affect real entities, enabling a concrete effectivity of modulations and becomings’. Therefore, the diagram draws together the vectors of modulation that affect an assemblage, directly relating to the futurity of an assemblage. But, as McCormack (2005: 124) writes,

although it is abstract, the diagram can nevertheless be apprehended as a real organisation of forces through the way it gives the relations between these forces a kind of spatiotemporal consistency. This notion of consistency can be understood as a mode of organisation that `holds together’ relations of force without ever precipitating or capturing these relations in the form of subject or object.

Deleuze and Guattari (1988: 142) explain that ‘the diagrammatic or abstract machine^4^ does not function to represent, even something real, but rather constructs a real that is yet to come, a new type of reality’.

In this way, a non-representational reading of a diagram can be set alongside work from architecture that comprehends diagrams and drawings as unfolding fields of potential new worlds (Jobst, 2013; Yaneva, 2012), or they can be read in the same way as diagrams in association football, not as visual representations of concrete actions, rather as animating devices that designate plans of action, formulations and movement that indicate the potentiality of bodies and space (Barnfield, 2016). Approached in this way, government funding frameworks and guidance documents can be seen as diagrams that aim to bring forth new realities.

#### Method: Urban development in central and devolved government in England

This article is drawn from phase 1 of the Tackling the Upstream Determinants of Unhealth Urban Development (TRUUD) project. The data in this article are from the section of TRUUD that has looked at urban development by exploring the interconnections of local and national government. An initial step for the whole project was to map the urban development system by working trans-disciplinarily to develop 10 key concepts to explore the problem space (Bates et al., 2023). Value was identified as a concept that is crucial to understanding urban development. This mapping work led to targeting specific interviewees who had experience or knowledge of the urban development system in England.

The findings of this article proceed from interviews (n = 24) with the key informants identified in the initial mapping work (senior civil servants in Whitehall department of Levelling Up, Housing and Communities (DLUHC) and Business, Energy, and Industrial Strategy (BEIS), politicians, local government representatives, third-sector actors) that took place during the summer of 2021. The interviews were on average 58 minutes in length, transcribed verbatim and analysed using NVivo 12. The NVivo analysis was conducted in a team format that developed an integrated codebook through weekly coding sessions and discussions to develop core themes (Braun and Clarke, 2019). The article also draws from an analysis of key urban development funding streams, UK Government documents, guidance notes and HMT’s investment rules. In this article, I use the term *public actor* to denote a civil servant or senior official from the UK Government, non-departmental public body or local government who have experience of administering, assessing, allocating or applying for funding for urban development in England. Due to anonymity requirements, only the departments and level of seniority of participants are mentioned in this article.

### Enacting value in urban development funding

The next section examines the tools and procedures of enacting value in urban development funding in England. It argues that HMT documents, guidance tools and appraisal rules, treasury ‘thinking’ and departmental processes establish the methods through which value is enacted in urban development funding and across government. This, in turn, helps to underpin the basis for urban development and the spatial-temporal grounds for local authority development and infrastructure projects.

#### Urban development funding

In England, local authorities play a key role in planning, developing and managing the built environment within their administrative boundaries (Built Environment Committee, 2016). Local councils develop a local plan, in which they outline their planning policies and allocate sites for different types of development. There are, however, national elements to this system. The local plan, before adoption, is required to show that it agrees with national planning policy and guidance. This guidance is issued by the Secretary of State (DLUHC, formerly MHCLG). Local plans are publicly examined by the Planning Inspectorate for England and Wales.

This means that local government plays a key role in shaping priorities and spatial developments. However, they do so while working inside the confines of a framework of national policy. Local government is required to respect the decisions of the national Planning Inspectorate. They also work with and respond to different national-level agencies, funders and decision makers. Therefore, urban development is resultant from national and local priorities, policies, negotiations and decisions. Urban development in England is funded through the allocation of money from central to local government (except national infrastructure projects like High-Speed Rail 2). It is through these funds that the central government can exert influence over what is built and where it gets built (Wu et al., 2022).

The funding streams available are vitally important for urban development (see Table 1 for key funding streams for urban development). The effects of austerity and the cuts to local government grants and budgets mean that the funding streams are essential for development (and some social services) in urban areas (Jupp, 2021). Local government and combined authorities bid for funding to carry out projects that include re-developments of high streets, new infrastructure and urban regeneration schemes. Some funding streams can be used for softer infrastructure projects such as skills and training programmes.

The funding streams are administered in different ways. For example, the Community Renewal Fund that is intended to replace EU Regional development funding is a competitive process. Designated lead authorities for each place invite bids from applicants for a share of that place’s funding pot. The guidance states that bids will be rated according to whether they align with wider investment priorities (MHCLG, 2021). In contrast, in the Towns Fund the government invited 100 places to develop proposals for a Towns deal. The process devised by DLUHC was to select towns and invite them to bid for funding.^5^ Taken together, the funds and policies indicate the government’s priorities and ambitions for Levelling Up. These ambitions could be tempered by existing government policy objectives and have implications for the intentions that have been identified in the Levelling Up white paper (HM Government, 2022).

#### The Green Book, value and investment

In spring 2020, a review of HMT’s Green Book^6^ was instigated by the government. This was in part due to growing criticism from leading political, civil and private-sector figures in Northern England arguing that the current guidance led to an overreliance on benefit-cost ratios (BCRs) in decision-making (Metro Dynamics, 2020). This resulted in a vicious cycle of a continual underdevelopment of strategic cases which in turn led to increased importance given to BCR by HMT officials and public actors (for a critique of BCRs, see Flyvbjerg and Bester, 2021).

The new Green Book seeks to overcome these shortcomings by assigning greater weight to applications making a strong strategic case. The requirement will now be for cases to unite projects to government objectives. This revision is significant because better developed strategic cases enable appraisers to assess the business case of each project based on factors other than the BCR. The strategic case must clarify which government strategic objectives are supported, in conflict, or constrained by legal limits on government intervention of any project. A prospective project must demonstrate how it fits into the four-tier objective hierarchy outlined in the Green Book: individual projects to a programme of projects to a strategic portfolio to a strategic objective (e.g. offshore wind farm to increasing wind capacity to reducing emission in power sector to net zero emission by 2050).

In terms of value, the new Green Book does not solely rely on BCRs. In a note to reviewers, HMT make it clear that they should

be open to a business case for projects with a low BCR if, compared to options that have been appraised, that option is the best value for money way of delivering an intervention that is necessary for the achievement of the intervention’s objectives. (HMT, 2020: 11)

The revision of the Green Book prepares the ground for the Government’s Levelling Up agenda by including place-based analysis: mitigating the regional impacts of policymaking with a focus on helping less well-off regions draw nearer to better off places. To achieve this, the Green Book (HMT, 2020: 91–96) outlines that if an intervention is in an area that is deemed in need, the benefits ought to be felt by that specific area and supports the application of distributional weights that accrue from funded projects to less well-off places (HMT, 2020: 54). An appraisal must either quantify benefits or explain why they are not relevant even if place-based effects are not a primary aim.

The Green Book hasn’t been the only effort that seeks to change how value is enacted by public actors. The Barber review, for HMT, introduced the public value framework in 2017 (Barber, 2017: 5–6). The recommendation of the review was to develop a concept of value from the public body to public spending. In introducing a framework for HMT, the idea was to foster an objective assessment of the value of spending programmes. In addition, the Public Services (Social Value) Act 2012 calls for all public-sector commissioning to take into consideration economic, social and environmental well-being in connection with public services contracts. The act requires that all public bodies in England and Wales (e.g. Local Authorities, NHS organisations) to examine how the services they commission and procure might improve the social, economic, and environmental well-being of the area.

The updated Green Book, funding guidelines, and evaluation criteria – taken alongside other government documents, for example, the guidance notes for health (HMT, 2013) and for well-being (HMT, 2021) – can be taken as an example of diagrammatic thinking. This is because they set out a framework that does not offer a set path for action. They are instead proposing a field for activity that as Deleuze explains should be seen as a rough outline of something to come. The Green Book and funding framework is not a structure; rather, it is an attempt to gather the continually unfolding forces that perpetually sweeps from the virtual^7^ to the actual to the virtual, continuously from territorialisations to deterritorialisations.^8^ As a set of relations of forces, it ‘never exhausts force, which can enter into other relations and compositions’ (Deleuze 2006 [1986]: 74).

In taking the Green Book and the associated funding framework as a diagram, two things happen. First, it emphasises the conditions of force and its influences as ‘it is a way of making power relations functions in a function, and of making a function through these power relations’ (Foucault, 1995 [1977]: 207). An influential force is rendered visible and spreads from one domain to another attempting to marshal new movements, new forces and new relations. In the case of urban development funding, the diagram shows that HMT is an influential force and to think about encouraging more lines of action, more lines of movement will entail drawing on more evidence, leaving more responsibility to other actors and other departments in central (and local) government. Second, it invites an opportunity to work back on that force, to open a space for urban development funding to be a field of experimentation drawing on new enactments of value that are open to the ever-changing continuous unfolding of flows of forces. Thinking diagrammatically shows that the ambitions of the government are encouraging changes to the structure of funding streams to bring about projects that meet local needs. However, the diagram exposes that this is done to engender political gains rather than offering a means for improving all places. The effect of this will likely be the redrawing of spatial inequalities between areas that are sensitive to the government’s view and those that are not (Connolly et al., 2021).

### Public actors view on value in urban development funding

Public actors are aware of how crucial central government funding is for urban development (Markusen, 2006). In England, the centralised policymaking structures and cuts to local government make funding essential. Money that used to be put directly to local government for urban development has been replaced by competitive bid funding. A senior public actor from DLUHC explained,

Within the urban decision making, we’ve got . . . somewhere in the region of 20 billion pounds through the Ministry for things such as homeless, cladding, town centre renewal and regeneration, monies towards the new settlements and such like. But a lot of that has to be bid for and is monies that perhaps 15 years ago would have gone straight to councils and be spent by councils but is now much more centralised as less monies are pushed out to local government.

The quote emphasises the importance of the central government funding streams for local authorities in England. It also demonstrates how the enacting of value is important to urban development because value is the result of institutions, policies, procedures and actors from the central government (Heinich, 2020). This indicates that the central government can retain control of the levers of policymaking and funding, which means that they can have a more direct influence on urban development. From the interviews with public actors working at a national, regional or local level in England, value was deemed to be enacted on terms influenced by HMT. The interviews brought up four core themes: (1) HMT is an influential force for how value is enacted; (2) change will be a slow, incremental process; (3) there is a recognised need to promote more than financial benefits; and (4) public actors are aware that to enact value differently changes to structures of decision-making need to be brought forth.

#### HMT is an influential force for how value is enacted

The enacted value is influenced by a range of different actors, processes and (devices). In urban development funding in England, HMT is an influential actor. This includes the way funding streams are designed and administered as well as the guidance documents and funding criteria. The desire for control over other departments has been suggested as an aim of HMT (Knights, 2022). The public actors added to this by highlighting how HMT was an influential voice at the table during decision-making. As a senior public actor from DLUHC outlined, the main influence on value is ‘the Treasury ultimately . . . they have quite a narrow concept of value for money’. This has led to specific ways of working. A senior public actor from BEIS described how there is a need ‘to find a way of making the argument in a way that Treasury will understand. I think that’s a big barrier’. This involved HMT focusing on returns on investment, business cases, and short-term time frames – issues that resonate with criticisms of the use of cost-benefit ratios (Flyvbjerg and Bester, 2021).

There are several ways that HMT influences the enacting of value. An important one is by setting guidance documents and procedures (as demonstrated in the previous section). In using specific tools that structure ways of working has instilled an institutionally enacted value. As a senior public actor from DLUHC outlined, projects in one centrally managed fund for urban development have to complete

a local assurance process for those projects that the local authority basically has to do. The Treasury Five Case Business Case Appraisal, and obviously we have said that should be proportionate to the size of the project. If it’s a 15-million-pound project, you need to do a bit better than if it’s a small one. In a few limited cases we’ve called in that business case for MHLG to agree.

The five-case model asks applicants to detail the strategic, economic, commercial, financial, and management case (HMT, 2018). Value is described as part of the economic dimension of the business case and should deliver the best value to the taxpayer. This involves demonstrating how well a project meets government spending objectives.

The influencing force of HMT to value filters through the system and means that departments and projects must speak their language. A senior official in the cities and local growth unit in BEIS stressed that HMT must be onside for a successful project: ‘if the Treasury don’t like something it’s rare it gets through, but not impossible. If a particular department has doubts over a project, you want to investigate why they have doubts over it’. An effect of the centrality of HMT on urban development is that different concepts of value and spatial variations cannot find a space to be included. A senior public actor in DLUHC outlined that

it sort of takes you back to that problem, the way in which The Treasury works in the first place. I think the Treasury would probably focus too much on the costs as opposed to the longer-term benefits of being able to . . . show how in different places these investments have made this difference and over time it has saved this, and over time it has resulted in this.

The narrow definition of value also works to structure relationships between government funding and private investment. Projects need to be attractive to private-sector funding to help receive HMT support, as a senior public actor in the cities and local growth unit of BEIS explained:

In terms of the decisions, we’re not necessarily looking for any particular types of projects. What we are looking for is a kind of, a clear strategic vision, and a set of proposals that appear to have a level of coherence with that vision and projects that look like they’re going to be viable and sustainable in helping places not only secure our investment, but potentially secure further [private] investment . . . is it forever going to be dependent on the funding bids, or sort of fall over as soon as our funding finishes basically.

An outcome of this is that private-sector investors often given the opportunity will predominately invest in projects that would return profit, which in turn leaves unfancied but vital developments or services as unattractive propositions (Mazzucato, 2011). It is also significant because it reinforces the idea that public-sector-funded services are a provision of last resort or only short-term deals.

In terms of influence, the treasury has force not only over funding decisions but also institutional sway over the likelihood that ministers or departments are able to work against the grain, as a senior Westminster official pointed out:

there are institutional issues around disaggregating out funding streams for particular areas and about providing what the Treasury considers to be appropriate accountability. I think those sort of process issues get in the way sufficiently to put Ministers off taking decisions.

The influence of HMT contrasts with how value is enacted in the private sector and by property developers where value is enacted by the interaction of sub-centres of control and influence (Brill, 2020). How these spheres meet poses interesting questions for development and overlapping enactments of value.

#### Change will be a slow, incremental process

As the policy actors interviewed identified, HMT is an influential force in defining value for urban development funding. The public actors referred to the updated government guidance documents that indicate a development within thinking at HMT. This shows that central government thinking is open to new ways of enacting value. A national public actor from a non-governmental body concerned with health outlined how this change can fit with a wider strategic outlook:

the treasury had a review of the green book it was to try and prepare the road for the levelling up agenda. But there was much more emphasis on the strategic intent of what you were trying at the very start of a process. Well actually I think the refresh of the green book was generally trying to get people to look at the strategic intent and the outcomes, social as well as economic of what you’re trying.

Changing guidance would mean that more places could benefit from central government funding. This should work against spatial restrictions and regional disparities. A senior public actor from DLUHC explained how a change in outlook should benefit some hard-to-fund projects. It is important to note that they comment that this was ‘a battle’ that was won from outside HMT:

I think increasingly so [that there has been a change]. There’s certainly more sympathy more generally for different kinds of evidence, and understanding that point that –well, things like the BCR battle that we’ve had with them. They kind of accept now that some of those rules make it impossible for certain places to ever be successful, and actually If you do want to level up, you absolutely have to, and it feels like they are sort of open to being more kind of creative, at least in principle.

This suggests that while HMT is an influential force in determining factors, it is open to thinking about value in different ways. However, the pace of change is slow and indicative of institutions and individual change being an incremental process (Wollmann, 2000). In the case of HMT and urban development funding, change does seem to be occurring from above and below. A senior public actor from BEIS outlined,

The Treasury is probably more hierarchical than anywhere, but it feels to us, and maybe that’s just the relationship we’ve got with officials, that they are kind of very much on our side and doing the best job they can to think about this stuff in different ways.

While interviewees felt that change was happening, current funding structures and procedures looked set to stay in place for some time. Introducing new ways for how money is awarded, administered and evaluated would be required for deeper change. A Senior Westminster official suggested that at present competitive bid-based funding with HMT guidance and rules is still the main method of distributing money to local authorities:

my impression is that the current state of government policy is very much around the large-scale funds which have been announced in the last twelve months . . . I’m not seeing any particular interest in doing things differently. I think that they’re very much, traditional, bid-based funds . . . there’ll be a process, money will be allocated, there’ll be accountability for that money – it’s a very traditional process.

#### A recognised need to promote more than financial benefits

The current funding guidelines and criteria have placed tight restrictions on funding projects that have more than financial benefits. A senior public actor in BEIS explained that there is an awareness across policy implementation that a space is required to consider the harder-to-monetize aspects:

There’s an undervaluation of some of these less tangible impacts. You have a kind of economic case around adding so many kilometres of cycle route, but actually it’s quite hard to convince the Treasury that it’s worth another five or six million to deliver health benefits. It’s just very hard really . . . we need to think about some of these longer-term sustainable development issues as part of the process of creating a value for money case for projects.

This would entail re-working the very notions of intangible and tangible. Health, for example, is not an intangible impact when considered in terms of ability to live as well as possible. It has implications for working, community involvement, and for society at large. Re-working intangible/tangible would lead to enacting value differently.

The position of HMT in the enacting of value in decision-making for funding streams prioritises short-term, financial accruements despite there being an awareness of the need to enact value with more regard to local temporal and spatial concerns. A senior public actor from the cities and local growth unit explained that ‘short-term incentives and the disconnect between costs versus long-term payoff. The coordination failures between [departments] over how to design urban policy when you’re reliant on some of the decisions that’s not been made’ are barriers to promoting more than financial benefits to investments in urban development.

The challenge to any re-working of value for policymaking is constrained by political situations. Political cycles influence the policy process. In England, general elections are every 5 years and local elections are every 4 years. This in combination with the recognised need for reducing regional disparities requires funding ‘soft’ infrastructure projects that need a different way of enacting value. A national public actor at a non-governmental body concerned with health outlined that

[t]he treasury have started the ball rolling there . . . the treasury has the money . . . to address those regional imbalances it’s about social capital as much as hard infrastructure. And the challenge of a government within a five-year parliamentary term is you can build things can’t you, so you navigate to the hard infrastructure. And then you can show voters you’ve built something, well that’s not going to tackle low pay, in work poverty, lack of skills.

In addition to funding soft infrastructure projects, public actors recognised the need to factor in for longer time scales in funding criteria to make certain projects more attractive. A senior public actor from DLUHC explained that

[w]e also need longer time horizons. There’s tendency towards just looking at each project consecutively in isolation a little bit, rather than genuinely having spatial [plan] – government doesn’t really have any great spatial sort of plans or priorities any more . . . doesn’t have that kind of integrated plan for development.

This would involve central government developing an integrated spatial plan that includes working with different layers of local and regional governance in England and include flexibility for urban development to meet the different needs of places.

A different way to enact value would need to filter throughout the urban development system because of the interplay between local and national competencies. Different interpretations of value can hinder what is developed, how developments are designed and how land is acquired or sold. A local government expert explained that

one of things about local authorities is . . . some of the key decisions they have to make. For example, public land disposal and getting good value for it. Local authorities differ quite significantly on their reading of what their obligations are in terms of things like, to what extent can they do anything other than sell it to the highest bidder . . . Clearly, if you go around the country, some local authorities would say, our reading the legislation is we can’t do anything other than sell it to [housing developer] or whoever because that gives us the most value for the taxpayer.

There are clear difficulties for local authorities, and other central government departments, to be able to fully engage with the technicalities of urban development funding. This is in part due to the way value is enacted and the lack of a clear working definition that can cope with the complexities of different situations (Robin, 2018; Weinstein, 2014).

#### Enacting locally sensitive value requires changing structures of decision-making

To enable urban development and associated funding to be more responsive to inequalities and regional disparities, public actors suggested that alongside a more sensitive conception of value, a broader development in how funding is allocated and how local and national priorities can fit together is required. The national public actor from a non-governmental body concerned with health explained that

If you look at the guidance for the towns deals, and the towns fund . . . is an example of a national funding pot, and this is the preferred mechanism for government now isn’t it, relatively small funding pots centrally co-ordinated to make it competitive. You could argue that places would rather have the money and the devolution to be able to make it but that’s a different decision.

A member of the House of Lords agreed, suggesting that competitive bid-based funding is a point of concern for local authorities and greater devolution is needed alongside more direct funding:

I think it’s a moment for real concern actually in terms of –and again, you know lack of devolution means that we’re subjected to bidding for pots of money. Obviously ((region)) has just become a mayoral authority which means we have access to some funding as of right, particularly around some of the transport funding.

The interviewees placed a lot of importance on value needing to have a place-specific appreciation and local authorities as arbiters. This would require more devolved powers to enable local and regional actors to be able to set local agendas, work collaboratively, and have the resources to shape spaces in ways that are sympathetic to local needs. The member of the House of Lords continued,

I think local authorities having a real important role as place shapers so that they can make sure that they have the partnerships that are needed around the table to assist with coming up with a development that should add value to the local area. And I think that’s probably where there’s a sense that in Britain, we’ve gone wrong. That developments just land with no connection.

Changes in structures of decision-making could facilitate different ways of thinking and working. At present, some of the challenges faced in answering difficult problems in urban development are due to reduced capacity. A senior public actor from DLUHC explained that a particular fund’s framework is intended to stimulate opportunities to foster more collaborative ways of working:

There’s an intervention framework that is very broadly drawn and it’s deliberately broadly drawn, because the intention of this is, it’s about urban development but it’s also very much about building capacity in places, it’s about building the things in places but it’s also about creating opportunities for different stakeholders within a place to come together, develop a shared vision, try some stuff out basically, and kind of see what results.

By exploring how value is operationalised in funding streams, the potential for different actors to be drawn into the processes and for capacity to be enhanced has been highlighted (McFarlane, 2021; Weber, 2016). The development of strategic alliances would be beneficial for projects and areas that may only accrue ‘intangible’ outcomes, but those outcomes would lead to improvements in daily life and new avenues for action.

### Enacting value: The implications for levelling up

The aim of this article was to understand how value is enacted in urban development funding. It has been drawn from interviews with public actors involved in funding and from UK government documents. Attending to how value is enacted reveals who influences actions and actors, which in turn influences how value is represented in decision-making and urban development projects. This article has added to the literature on performativity of value in real estate by exploring how value is enacted by public actors in urban development funding (Brill, 2020; Robin, 2018). It has added to the growing literature on value by examining how value is enacted through institutions, procedures and funding mechanisms (Heinich, 2020).

In England, HMT is influential to how value is enacted via urban development funding. The prevailing conception of value in urban development prioritises short-term, financial outcomes despite an awareness of the need to be sensitive to temporal and spatial concerns. How this force connects to other forces like private-sector influences or flows of international investment is worth detailed investigation. Value is conceptualised and operationalised within central government funding with regard to the rules and guidance set by HMT.

The guidance documents, evaluations of bids, and development of a business case in line with expectations work to structure decision-making and thinking for urban development funding for local authorities. This is significant because it is applications and approved projects that determine what is built, what sort of projects are finalised, and how urban space is developed. It also informs urban imaginaries, project development ideas, and relationships between forms, styles, and materials of construction (Amin and Thrift, 2017). The public actors interviewed recognised that the current rules and obligations made it difficult for certain places and certain types of projects. As the government documents show, HMT has recognised the need to think about value differently (HMT, 2020).

The concept of the diagram provides a critical way of apprehending the spatial and political dimensions of urban development funding. Approaching funding guidelines and guidance in this way has revealed the forces and dynamics that influence process and procedure. Diagrammatic thinking suggests that there are multiple lines of flight, some of which are actualised, and some not. Aside from exposing influential forces, the diagram suggests the need to find a way for an imaginative engagement with funding objectives and strategic outcomes, one that seeks to work back on such forces and open a space for urban development funding to be a field of experimentation. This would entail establishing different enactments of value that can make more of spatial and temporal concerns, looking for different types of evidence or seeking to engender outcomes that are not circumscribed by financial results.

The way value is (re)produced by public actors and institutions shapes everyday geographies in fields other than urban development and funding. It is imperative to consider the different ways value overlaps public and private actors with the latter working with and against the former. Thus, there is a need to identify the different actors who define value and how those definitions shape urban space. This would be an additive to recent work that has looked at financial geography and how processes of financialisation shape cities and municipal activities (Aalbers, 2019; Weber, 2021). Future research should examine how value is enacted within HMT, Government Economic Service, and the Government Economic and Social Research team. These parts of HMT influence how value is enacted by other government departments and non-government organisations. How this intersects with private-sector actors and other private institutions is also an important avenue of future research.

How value is enacted has clear implications for the UK government’s Levelling Up agenda. The levelling up white paper, published in February 2022, sets out the intention of the government to address long-standing regional disparities. Through 12 missions, it aims to reduce spatial disparities by providing the groundwork for place-based solutions via targeted funding, better use of data and some devolution (HM Government, 2022). This is significant for two reasons. First, it does indicate that government thinking is open to, and actively pursuing, new ways of thinking, understanding and working with concepts like value. Second, to be sensitive to the needs of different places more than financial benefits will need to be brought into funding allocations and decision-making structures. Health is a good example and would involve bringing together voices that are often left out of discussions of urban development. In establishing health outcomes into funding streams and allocations, long-term perspectives could encourage more sensitive development and would help to re-orientate thinking to be cognisant of locally specific issues.

A key piece of levelling up involves thinking about value and how it can be employed in different places. The need for enacting value with consideration for regional disparities is clear from the interviewees in this article and the inequalities that are present throughout England. Such inequalities are found between and within regions and cities. Encouraging decision-making to be devolved from the highly centralised state is important for meeting the demands of the country and the objectives for the levelling up agenda. Value and how it is enacted should be central to efforts to levelling up because to work effectively, it requires a concept of value that can be enacted by all actors from public and private sectors in their efforts to develop urban space and work towards reducing spatial inequalities. Levelling up requires devolution across and within the central government as well as down and out towards local government and non-government actors. This would entail broadening the forces that are influential in enacting value, to embrace more than HMT rules and procedures. Tackling the influence of HMT would be a bold start for Levelling up and one that could prove crucial for urban development.

## Figures and Tables

**Table 1:** UK Government funding relevant for urban development (source: Centre for Cities/UK Government).

Funding stream	Description
*Levelling Up Fund*	A £4.8 billion fund for ‘shovel-ready’ infrastructure projects across the United Kingdom. This money will be spent up to 2024/25. Bids for the first round of funding were submitted between March and June 2021, and final decisions were made in autumn 2021.
*Towns Fund*	A £3.6 billion fund for towns in England, including money for the regeneration of high streets. A total of 101 towns were eligible to bid for up to £25 million each for Town Deals, with the allocation of funding announced in July 2021. Funding will predominantly be for investment, focusing on urban regeneration, planning and land use; skills and enterprise infrastructure; connectivity.
*Community Renewal Fund*	A £220 million fund for the whole of the United Kingdom in 2021/22, which aims to provide a bridge between EU structural funds and the planned new UK Shared Prosperity Fund (UKSPF). Most of the funding is revenue funding, with a small amount of capital funding. Projects must align with at least one of the following priorities: skills; supporting people into employment; local business; communities and place.
*Freeports*	Low-tax and low-tariff business zones. Locations bid to become freeports and eight were selected and announced at the March 2021 budget.
*Skills Fund*	A £2.5 billion fund for adult skills, including a guarantee of free access to level 3 qualifications and funding for skills boot-camps.
*UK Infrastructure Bank*	A new government-owned bank that will be able to finance infrastructure projects. The bank will have £12 billions of capital for lending and investment, and the authority to issue up to £10 billion of guarantees.
*Civil service relocation*	Movement of civil service jobs away from London and the south-east of England, with a target to move 22,000 jobs by 2030.

UKSPF: UK Shared Prosperity Fund.

## Notes

1 A combined authority is a legal body set up using national legislation that enables a group of two or more councils to collaborate and take collective decisions across council boundaries (see https://www.local.gov.uk/topics/devolution/devolution-online-hub/devolution-explained/combined-authorities).

2 His Majesty’s Treasury is the department of the Government of the United Kingdom responsible for developing and executing the government’s public finance policy and economic policy.

3 Levelling Up is suggested to be a whole of government approach for economic and social programme that aims to reduce entrenched regional disparities and boost productivity, growth, and prosperity throughout the United Kingdom. The Levelling Up fund is part of this effort and is designed to invest in infrastructure that improves daily life across the United Kingdom.

4 An abstract machine is a diagram independent of the forms and substances, expressions, and contents it will distribute. They are virtual multiplicities which do not exist independently of the assemblages in which they are actualised or expressed (Patton, 2000).

5 The Towns Fund has been criticised by the Public Accounts Committee. The Committee’s report called the process ‘not impartial’ and criticised the role of ministers in selecting towns (see https://publications.parliament.uk/pa/cm5801/cmselect/cmpubacc/651/65105.htm#_idTextAnchor002).

6 The green book provides ‘technical guidance’ to help officials advise ministers how best to achieve a given policy objective. It does not set policy objectives. As ultimate decision makers, ministers are not bound by recommendations arising from green book appraisals (House of Lords Library https://lordslibrary.parliament.uk/government-investment-programmes-the-green-book/).

7 The virtual is an aspect of reality that may not be real in a physical sense, but nonetheless real: ‘The virtual is opposed not to the real but to the actual. The virtual is fully real in so far as it is virtual’ (Deleuze, 2014 [1986]: 272).

8 Deterritorialization is the occurrence by which a social relation, defined as a territory by Deleuze and Guattari, has its current organisation and context altered, mutated or destroyed.
